# Real-life study to assess effectiveness and safety of eculizumab in patients with neuromyelitis optica spectrum disorders in France: protocol for ECUP4, an observational study

**DOI:** 10.3389/fneur.2023.1303874

**Published:** 2024-01-22

**Authors:** Romain Marignier, David Laplaud, Hélène Zéphir, Caroline Papeix, Emmanuelle Leray, Ekbel Amri, Mickaël Piotaix, Jérôme de Sèze

**Affiliations:** ^1^Service de Neurologie, SEP Neuroinflammation et Pathologies de la Myéline, Centre de Référence des Maladies Inflammatoires Rares du Cerveau et de la Moelle (MIRCEM) - Hôpital Pierre Wertheimer, Bron, France; ^2^Service de Neurologie du CHU de Nantes, CR2TI-Inserm U1064, CRC-SEP Pays de la Loire, Nantes Université, Nantes, France; ^3^Service de Neurologie du CHU Hôpital Roger Salengro, Lille, France; ^4^Service de Neurologie, Hôpital Fondation A. de Rothshchild, Paris, France; ^5^Univ Rennes, EHESP, CNRS, Inserm, ARENES UMR 6051, RSMS U 1309, Rennes, France; ^6^Alexion, AstraZeneca Rare Disease, Levallois Perret, France; ^7^Service de Neurologie du Centre Hospitalier Universitaire de Strasbourg, Strasbourg, France

**Keywords:** neuromyelitis optica spectrum disorders, eculizumab, real-world evidence, complement inhibition, disease-modifying therapy

## Abstract

**Background:**

Eculizumab, a humanized monoclonal antibody targeting the C5 complement protein, has been approved for the treatment of neuromyelitis optica spectrum disorders (NMOSD) in adult patients who are anti-aquaporin-4 (AQP4) antibody positive (Ab+). The aim of this study is to evaluate the long-term effectiveness and safety of eculizumab in French adults with NMOSD and to describe patients' characteristics, disability, and quality of life using data collected in a real-world setting.

**Methods:**

This is the protocol for ECUP4, an ongoing prospective, observational, non-comparative, multicenter study conducted in 32 reference centers in France. Eligible patients must also be enrolled in NOMADMUS, a nested cohort of the French national multiple sclerosis registry (OFSEP). The primary endpoint is the annualized relapse rate. Secondary endpoints include the long-term safety of eculizumab, as well as patients' characteristics, treatment outcomes, disability, pain, visual acuity, and quality of life. Visits and treatments follow routine clinical practice. The case report forms (CRF) comprise data recorded in the context of the NOMADMUS cohort, collected during routine visits. The inclusion period is planned for 3 years, with no limitation on the number of patients enrolled. The maximum follow-up duration will be 5.5 years.

**Conclusion:**

The efficacy and safety of eculizumab in patients with AQP4+ NMOSD have been demonstrated in randomized clinical trials that showed a significant reduction in the risk of relapse, with a safety profile consistent with other indications. This study will provide clinical and patient-reported evidence of the benefits of eculizumab, using data from a real-world setting in France.

**Trial registration number:**

This study is registered at the French public repertory Health data Hub, N° F20211228123801. All information can be accessed at: https://www.health-data-hub.fr/.

## Introduction

Neuromyelitis optica (NMO), initially described by Devic, was clinically characterized by a severe episode of optic neuritis and transverse myelitis (1, 2). The discovery of autoantibodies directed against the aquaporin-4 (AQP4) astrocyte water channels led to the concept of Neuromyelitis Optica Spectrum Disorders (NMOSD) for seropositive patients, in whom typical NMO, isolated optic neuritis (ON), or inaugural longitudinally extensive transverse myelitis (LETM) could coexist with or even be preceded by other clinical presentations (3). Approximately 73% of NMOSD patients are AQP4-seropositive. Subsequently, it has been acknowledged that some patients were seronegative for AQP4 antibodies, while anti-myelin oligodendrocyte glycoprotein (MOG) antibodies could be detected or not (double seronegative cases) (1). Among patients with AQP4-IgG-negative disease, 42% were positive for anti-MOG antibodies (4). The current definition of NMOSD was established in 2015 by an international panel of experts that was convened to develop revised diagnostic criteria and clarify the nomenclature of the disease (3). The core clinical characteristics requirements for patients with AQP4-IgG-positive NMOSD include syndromes related to the optic nerve, spinal cord, area postrema, other brainstem, diencephalic, or cerebral presentations. More stringent clinical criteria, with additional neuroimaging findings, are required for the diagnosis of NMOSD without AQP4-IgG or when serologic testing is unavailable (3). AQP4-antibody seropositive disease is considered primarily an autoimmune astrocytopathic disease with lytic and non-lytic clinical consequences for astrocytes, while MOG-antibody seropositive disease is an inflammatory demyelinating disease associated with a better prognosis (1, 5). The worldwide prevalence of NMOSD is estimated at around 1–3 per 100,000 people. NMOSD is more frequent in women, with a female-to-male ratio up to 9:1 and an age at onset of 40 years on average (6). In France, 669 NMOSD patients with AQP4-IgG+ were included in the NOMADMUS cohort by April 2022. The age at onset was 41 years on average, and a similar female-to-male ratio was observed in this cohort.

In NMOSD, relapses are more frequent (90%) (7) and more severe (8), and prognosis is generally poorer with potentially higher Expanded Disability Status Scale (EDSS) scores (2) compared to multiple sclerosis. NMOSD is a severe and debilitating disease, the evolution of which is mainly driven by the occurrence of relapses. Relapses may lead to vision loss and paralysis, often only partially reversible, and this is also in contrast to MS, where relapses generally involve full recovery (9). The proportion of patients with complete remission inversely correlates with the number of attacks (10), and neurological deficits accumulate over time. Patients with relapses exhibit diminished visual acuity, clinically meaningful worsening of neurological disability, and greater use of analgesics use than non-relapsing patients (11). Quality of life is significantly reduced in patients with NMOSD, and most patients report impairment in several domains (12). Thus, the burden of disease is particularly high, and every effort must be made to reduce the incidence of relapses since they are closely associated with disease progression.

Treatment of NMOSD comprises three components: acute treatment of relapses, preventive therapy, and symptomatic therapies. The mainstay of acute treatment consists of corticosteroids and plasma exchange (PLEX). A recent retrospective study using data from NOMADMUS suggested that aggressive anti-inflammatory treatment requiring an early combination of PLEX with corticosteroids for the first attack is associated with better recovery and treatment outcomes (13). Preventive therapy is aggressively pursued through immunosuppressants (14). It relies on off-label use of immunosuppressive drugs, commonly azathioprine, mycophenolate mofetil, and rituximab or immunoglobulins in patients with contraindications to immunosuppressants (14, 15). Nevertheless, new approved therapies are emerging in the NMOSD treatment strategies, such as satralizumab, inebilizumab, eculizumab, and ravulizumab. In France, specific guidelines for the management of NMOSD were published in 2021 (16).

Eculizumab is a recombinant humanized monoclonal IgG2/4k antibody that binds to the human C5 complement protein and inhibits the activation of terminal complement. Eculizumab was the first drug to be approved for the treatment of AQP4+ NMOSD, based on the results of the phase III PREVENT study (ClinicalTrials.gov; PREVENT: NCT01892345; open-label extension: NCT02003144) that demonstrated a statistically significant reduction in the risk of adjudicated relapse by 94% compared with placebo (*p* < 0.0001) in patients with AQP4-IgG+ NMOSD. Eculizumab safety data were also consistent with its well-characterized safety profile, with the specific risk of meningococcal infections in its other approved indications, paroxysmal nocturnal hemoglobinuria (PNH), atypical hemolytic uremic syndrome (aHUS), and generalized myasthenia gravis (gMG), based on clinical trial data and more than 10 years of post-marketing experience (17–22). Eculizumab was granted in 2019 a marketing authorization for the treatment of adult patients with NMOSD who are AQP4 antibody-positive with a relapsing course of the disease by the European Medicines Agency (EMA). In France, the Health Authority, *Haute Autorité de Santé* (HAS), Transparency Commission issued a favorable opinion on reimbursement of eculizumab in adults with NMOSD AQP4+ and with a relapsing course of the disease (two relapses in the past year or 3 relapses in the past 2 years, including one in the past year) and not responding to background immunosuppressant therapy (rituximab, azathioprine, mycophenolate mofetil) (23). To further assess the benefits of eculizumab in post-marketing surveillance, the French authorities required an observational study to evaluate the long-term effectiveness, safety, and patient-reported outcomes of eculizumab in patients with NMOSD in a real-world setting.

## Methods

### Study design and setting

ECUP4 is a prospective, observational, non-comparative, multicenter study, performed at highly specialized French sites part of the network MIRCEM (*Maladies Inflammatoires Rares du Cerveau et de la Moëlle*) or being centers of resource and competency for multiple sclerosis CRC-SEP (*Centres de Ressources et de Compétences sur la Sclérose en Plaques*). Approximately 32 sites in total are included. Patients enrolled in ECUP4 must also be included in NOMADMUS, a nested national cohort of the French multiple sclerosis registry. NOMADMUS is a prospective, multicenter, observational study of patients with NMOSD, and related disorders in France (clinicaltrials.gov NCT02850705) (24, 25). In this cohort, prevalent cases (previously diagnosed NMOSD patients) are included retrospectively and then followed prospectively over time; incident cases (newly diagnosed NMOSD patients) are included from the date of first diagnosis and followed prospectively. A minimal set of data has been defined and synthesized on specific forms derived from the European Database for Multiple Sclerosis (EDMUS) forms. However, additional data are captured to address the objectives of the current study. All the data are centralized in an EDMUS-derived database using the EDEN software. All cases are systematically tested regarding their AQP4-IgG and MOG-IgG status and validated by an expert committee. Participation in the study does not impact the usual management of the disease or the type of collected data since the case report forms (CRF) used in this study include data recorded in the context of the NOMADMUS cohort during routine visits. Patients can leave the study at any time for any reason, with no influence on their quality of care.

### Objectives

The primary objectives are to evaluate the long-term effectiveness of eculizumab based on the annualized relapse rate among NMOSD patients. Secondary objectives include the long-term safety and tolerability of eculizumab, describing the impact on disability and quality of life and understanding how eculizumab is prescribed to patients with NMOSD in real life. The study design has been established and approved by an independent scientific committee.

### Study population

All patients followed in participating sites, aged at least 18 years, diagnosed with NMOSD according to Wingerchuk criteria 2015 (3), and scheduled to start eculizumab or having started eculizumab <6 months prior to inclusion are eligible. All patients must sign an informed consent form prior to any study activity. Only patients with contraindications to eculizumab cannot be included in the study. Therefore, the eligible population is expected to be representative of French patients treated with eculizumab in real life. Patients treated with eculizumab receive 900 mg weekly for the first four doses. Starting the fifth week, patients receive a maintenance regimen of 1,200 mg every 2 weeks. All patients are required to receive meningococcal vaccination in the 5 years prior to, or at the time of, eculizumab initiation. Patients' characteristics include the following information: demographics, comorbidities, NMOSD history, including the presence of anti-AQP4 and anti-MOG antibodies, the number and annualized rate of relapses before inclusion, the date of the last relapse, prior therapies, and baseline clinical examination data.

### Schedule of assessments

**Figure 1:**
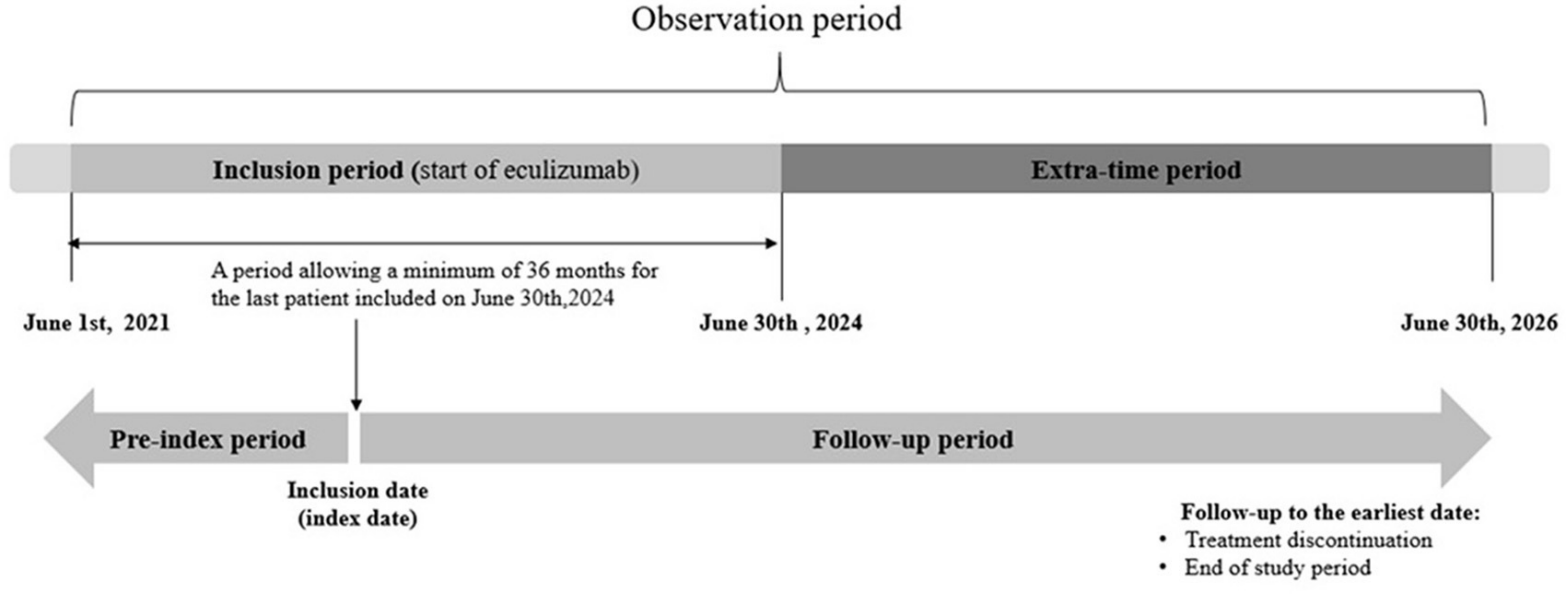
Overall study design.

Patients will be followed according to the usual clinical practice. No additional examination or assay is required as per protocol. Given the rarity of the disease, a 3-year recruitment period has been set to include a larger set of patients. Individual follow-up will be comprised between 6 and 66 months. Patients' participation will end at the time of study termination or 6 months after the last eculizumab administration in the case of premature treatment discontinuation, whichever occurs first. The overall study design is shown in Figure 1.

### Study duration

As the first patient was included in June 2021, recruitment will be terminated on 30 June 2024 and follow-up will end on 30 June 2026.

### Study endpoints

The primary endpoint is the annualized relapse rate. Relapse is defined as the occurrence of any new neurological symptom or worsening of existing symptoms with new abnormalities at neurological examination, persisting for more than 24 h, in patients with stable disease for at least 30 days. Symptoms must be attributed to NMOSD, and other causes (infection, excessive physical effort, or increased temperature) must be ruled out. An isolated modification of MRI or other imaging techniques without clinical modifications is not considered a relapse. Relapse confirmation will be based on a neurologist's assessment, and abnormality MRI findings are not required for the diagnosis. Relapses occurring during the study will be thoroughly described: type of relapse [all syndromes, syndromes having required treatment, ON, transverse myelitis (TM), ON + TM, area postrema syndrome, encephalic or diencephalic syndrome, and acute brainstem syndrome], hospitalization, MRI results, treatment, and recovery outcomes.

Secondary effectiveness endpoints include number of patients with at least one relapse during follow-up, time from inclusion to first relapse, evolution of annualized relapse rate by 1-year period, and changes in various clinical scores, namely, Kurtzke EDSS score (26), 7.5-m walk test, Nine Hole Peg test (NHP test), visual acuity evaluated by the number of letters read by each eye at Snellen test, and pain evaluated by visual analog scale.

The EDSS scale ranges from 0 to 10 in 0.5-unit increments with an increasing level of disability in eight functional domains, namely, pyramidal, cerebellar, brainstem, sensory, bowel and bladder, visual, cerebral, and others. For the 7.5-m walk test, patients are instructed to walk rapidly along a 7.5-m line one-way and return. The time to walk the distance is recorded (or the inability to pass the test if the patient cannot walk 7.5 m).

The NHP test is used to assess fine manual dexterity. It is composed of a square board with nine pegs. At one end of the board are holes for the pegs to fit in, and at the other end is a shallow round dish to store the pegs. The patient is required to take the pegs from a container, one by one, and place them into the holes on the board as quickly as possible, then remove the pegs from the holes, one by one, and put them back into the container. In the Snellen chart, each row of letters represents the minimum size of letter that a person with normal vision would be able to see at 6 m, 9 m, and various intervals up to 60 m.

Quality of life will be assessed using the European Quality of Life-5 dimensions (EQ-5D-5L) and 36-item short-form health survey (SF-36) scores. EQ-5D is a broadly used generic multi-attribute health utility instrument, comprising a visual analog scale (VAS) ranging from 0 (worst imaginable health) to 100 (best imaginable health) and a descriptive system of five dimensions with one item per dimension: mobility, self-care, usual activities, pain/discomfort, and anxiety/depression (27). SF-36 is a set of generic, easily administered quality-of-life measures comprising eight scales: physical functioning, role physical, bodily pain, general health, vitality, social functioning, role emotional, and mental health. Two distinct concepts are measured: a physical dimension, represented by the Physical Component Summary (PCS), and a mental dimension, represented by the Mental Component Summary (MCS).

Treatment outcomes include modalities of eculizumab use (posology, planned schedule of infusions, total duration of exposure, number of treatment discontinuations, and reasons for discontinuation), compliance (number of infusions between visits), prophylaxis of *Neisseria meningitis* infections (time from vaccination to first eculizumab infusion, prophylaxis including antibiotic agent, dosing regimen, and duration), concomitant therapies (start and discontinuation of rituximab, azathioprine, mycophelolate mofetil and corticosteroids, and analgesics consumption), and management of relapses, including hospitalizations.

Safety endpoints include incidence of adverse events (AEs), serious AEs (SAEs), treatment-related AEs and SAEs, AEs leading to treatment discontinuation, and AEs of special interest (AESIs), including infections due to *N. meningitis* or *Aspergillus* spp. which are classified as SAE, sepsis, and infusion-related reactions. Annualized rates of SAEs and AESIs will be provided.

### Sample size

Given that ECUP4 is an observational study with no planned comparison, no formal calculation of sample size has been performed, and there is no defined restriction on the number of patients to be included. Based on the sites' feasibility, it is expected that a total of 100 patients can be enrolled during the 3-year recruitment period. Assuming that the inclusion rate will be consistent over the years, ECUP4 is expected to collect data on 245 patients per year for the study period with a maximum follow-up of 4 years. In the PREVENT study, the annualized relapse rate in the eculizumab arm was 0.016 (95% CI 0.005; 0.5) per patient-year (28), which would correspond to four relapses in all patients. In ECUP4, the estimated confidence interval of the incidence rate, according to a Poisson model, would therefore be [0.006; 0.043] per patient per year.

### Data collection and management

Data will be collected during routine visits until the end of the study or 6 months after the last eculizumab administration, whichever occurs first. The schedule of visits is not required by a defined protocol but is decided by investigators as per clinical practice and French guidelines (16). Patients usually see their neurologist once or twice a year or when a relapse occurs. Quality of life will be evaluated by self-administered questionnaires that patients will fill out every 6 months or during a follow-up visit if it takes place more than 6 months after the previous one. Pain will be assessed at each visit. Since patients included in ECUP4 must also be included in the NOMADMUS cohort as required by the HAS, the tool used to collect NOMADMUS data will be adapted for data collection in ECUP4. The list of data collected at inclusion and follow-up visits is displayed in Table 1. EDMUS Services SAS is responsible for clinical operations including site management, remote and on-site monitoring, quality control, data management, and statistics.

**Table 1:** List of data collected at inclusion and follow-up visits.

	**Inclusion visits**	**Follow-up visits**
**Demographic characteristics**		
Age at inclusion	x	
Gender	x	
**Relevant medical history**		
Comorbidities	x	
Auto-immune diseases	x	
Malignancies	x	
**History of NMOSD**		
AQP4-IgG status	x	
MOG-IgG status	x	
Date of last relapse prior to inclusion	x	
Prior medication	x	
Evolution of NMOSD during follow-up		
Relapse since last visit		x
Type of relapse		x
Acute therapies for relapse		x
Hospitalization		x
Recovery		x
Clinical status		
Ambulation	x	x
Functional scores (26)	x	x
EDSS (26)	x	x
7.5-m walk test	x	x
NHP Test	x	x
Pain	x	x
Visual disability	x	x
Visual acuity in high and low contrast (Snellen test)	x	x
EQ-5D-5L	x	x
SF-36	x	x
Brain and spine MRI	x	x
**Use of eculizumab**		
Dosage and schedule of infusion		
Antibioprophylaxis	x	x
Prophylaxis of *Neisseria meningitis* infections	x	
Discontinuation of eculizumab		x
Use of concomitant therapies		x
Concomitant therapies	x	x
Pain medications	x	x
**Safety data**		
Any adverse events		x
Serious adverse events		x
Adverse events related to eculizumab		x
Adverse events of special interest		x

### Statistical analysis

Only descriptive statistics will be performed. Continuous variables will be summarized using means, standard deviations, medians, ranges, and interquartile intervals. Categorical variables will be summarized using counts and percentages. The duration of follow-up for each patient is defined as the time from the first infusion of eculizumab to the last visit under treatment.

The annualized relapse rate will be estimated by a Poisson regression model using the logarithmic function of follow-up duration as an offset term. In the case of overdispersion, a negative binomial regression model will be applied. Time-to-event variables will be displayed using the Kaplan-Meier curves. The evolution over time of EDSS, the 7.5-m walk test, and visual acuity will be described using mixed models for repeated measures. Missing data will not be imputed. Statistical analysis will be performed using the SAS and R software.

An interim analysis is planned for March 2025 to provide a report to the Haute Autorité de Santé in September 2025 as required. It will include all available data, with a focus on patients' characteristics, effectiveness, safety, and quality of life.

## Discussion

ECUP4 is a real-world study that will investigate the long-term benefits of eculizumab in NMOSD patients in France. The main purpose of the study is to evaluate the long-term effectiveness and safety of eculizumab in real-world use and to capture patients' perspective toward treatment outcomes. The study was planned in the context of a post-marketing surveillance request by the French authorities. The ECUP4 study is nested in the national registry NOMADMUS, which will ensure the quality and reliability of the collected data and will provide evidence for medical decision-making in treating NMOSD patients.

The rationale for the use of eculizumab in NMOSD was based on the evidence that complement activation with extensive vasculocentric immune complex deposition has been identified as one of the pathophysiological features of the disease (29). AQP4 antibodies trigger the complement system and lead to the formation of a membrane attack complex via the complement-dependent cytotoxicity pathway, which results in astrocyte damage and secondary neuronal injury (29). Eculizumab inhibits terminal C5 complement protein cleavage into the C5a and C5b fragments. It demonstrated its efficacy in the phase III PREVENT study, where 143 patients were randomized in a 2:1 ratio to receive either intravenous eculizumab (at a dose of 900 mg weekly for the first four doses starting on day 1, followed by 1,200 mg every 2 weeks starting at week 4) or matched placebo (28). Adjudicated relapses occurred in 3 and 43% of patients in the eculizumab and placebo arms, respectively, resulting in adjudicated annualized relapse rates of 0.02 and 0.35, respectively (rate ratio: 0.04; 95% CI: 0.01–0.15; *p* < 0.001). More recently, an interim analysis of combined data from PREVENT and its open-label extension, performed at 192 weeks (3.7 years), showed that 94.4% of patients treated by eculizumab remained adjudicated relapse-free. The adjudicated annualized relapse rate was 0.025 (95% CI: 0.013–0.048) in all eculizumab-treated patients vs. 0.350 (95% CI: 0.199–0.616) in the placebo group (30). Importantly, during the open-label extension phase, 37% of patients could stop or decrease their background immunosuppressive therapy use.

An indirect comparison of newly approved drugs using a Bayesian network meta-analysis suggested that eculizumab was more efficient than satralizumab or inebilizumab to reduce the risk of relapse, which was decreased by 90% compared to satralizumab and by 89% compared to inebilizumab, all drugs being used in monotherapy (31). Whether used as a single agent or combined with immunosuppressive drugs, eculizumab was associated with the highest probability (>80%) of being the best option to prevent relapses.

Eculizumab has also been approved in other indications: PNH, aHUS, and gMG. In these patient populations, it demonstrated a favorable safety profile. Socié et al. (21) published the largest safety data representing more than 10 years of post-marketing pharmacovigilance surveillance of eculizumab for the treatment of PNH and aHUS and confirmed that the overall safety profile of eculizumab is consistent with that reported from clinical trials. The major risk of eculizumab treatment remains the risk of neisserial infections, and there is a need for appropriate monitoring to effectively mitigate the risk of meningococcal infection (21).

Real-world data are increasingly used to obtain additional information, complementary to that provided by randomized controlled trials (RCTs) (32). One of the key advantages of real-world studies is that they enlarge the patient population compared to RCTs that require homogenous cohorts and exhibit, therefore, low external validity and generalizability to other settings (33). Follow-up is usually limited to relatively short periods in RCTs, external parameters are thoroughly controlled, and patients' monitoring is very stringent, which yields results that are reliable but poorly applicable in the overall population (32–34). Real-world data are therefore a very useful tool for decision-making, not only at the physician level but also at the regulatory level (34). The European Medicine Agency (EMA) and the Food and Drug Administration (FDA) issued recommendations to encourage pharmaceutical companies to perform real-world studies and provide real-world data as complementary information in their marketing authorization application files, or after drug approval, to allow reevaluation of the benefit-risk ratio (35, 36). In France, the HAS published guidelines on the methods to be used in post-marketing studies (37). The results of this study will add evidence to supplement results from clinical trials PREVENT on the effects of the complement inhibitor eculizumab and its safety profile. This might also help to inform the discussion of benefit and risk in the shared decision-making process for relapse prevention between healthcare providers and individual NMOSD patients. Currently, a post-marketing surveillance study is being conducted in Japan, including 179 NMOSD patients treated with eculizumab, which may last up to 7 years (38).

The main aspect of the study lies in the expected nationwide coverage of patient recruitment. To reduce any bias in patients' selection, inclusions must be as large and exhaustive as possible. In France, patients with rare diseases are usually managed in reference centers as part of a reference network. In this study, all possible sites identified as treating patients with NMOSD are involved in the MIRCEM network and CRC-SEP, which allow an almost comprehensive inventory of eligible patients. Nevertheless, some patients treated outside of these sites might be missed. In this study, the utilization of a well-defined NMOSD cohort with high diagnostic certainty needs to be acknowledged. All included patients have been positively tested for AQP4-IgG, and their diagnosis has been confirmed by expert neurologists. The attacks are commonly confirmed by MRI, enabling a precise measure of relapse. However, it is possible that some attacks are missed or not confirmed by MRI in the real world. The planned follow-up period will be potentially sufficient to provide long-term data on safety and effectiveness in this indication, extended for 5.5 years.

Some other aspects of the study deserve consideration. Since patients who started eculizumab <6 months prior to inclusion are eligible for the study, some historic data at baseline will be recorded retrospectively in case report forms, a situation considered a possible source of bias. However, follow-up data in the NOMADMUS cohort will be collected in a prospective manner using high-quality procedures, with few missing data points. Moreover, the classification of relapses will reflect real-world practice; thus, a classification bias cannot be fully ruled out. Yet, only highly specialized sites will participate, where physicians have long-lasting experience in patients' assessments.

Finally, new drugs are emerging in NMOSD. Ravulizumab is a second-generation humanized monoclonal antibody that binds to C5 and was developed to reduce the treatment burden associated with eculizumab through an improved dosing regimen. It provides the same benefits as eculizumab but has a substantially longer half-life (50 vs. 11 days), thereby permitting a longer intravenous dosing interval (every 8 vs. every 2 weeks). The efficacy and safety of ravulizumab were evaluated in a phase 3, open-label, externally controlled interventional study, CHAMPION-NMOSD (NCT04201262) (39). The PREVENT placebo group was used as an external comparator. Results showed that ravulizumab significantly reduced relapse risk in patients with AQP4+ NMOSD. Ravulizumab was recently approved by the EMA, as well as by the Brazilian and Japanese regulatory authorities. Once ravulizumab is available in France, it is expected that patients treated with eculizumab will switch to ravulizumab to reduce the treatment burden. Therefore, the number of patients to be enrolled in ECUP4 might be limited by the approval of ravulizumab in France.

## Ethics statement

The study was approved by Commission Nationale de l'Informatique et des Libertés. The study was conducted in accordance with the local legislation and institutional requirements. Written informed consent for participation was not required from the participants or the participants' legal guardians/next of kin in accordance with the national legislation and institutional requirements.

## Author contributions

RM: Conceptualization, Investigation, Methodology, Supervision, Validation, Writing – review & editing, Project administration. DL: Writing – review & editing, Conceptualization, Investigation, Methodology, Supervision, Validation. HZ: Conceptualization, Investigation, Methodology, Supervision, Validation, Writing – review & editing. CP: Conceptualization, Investigation, Methodology, Supervision, Validation, Writing – review & editing. EL: Investigation, Methodology, Software, Supervision, Validation, Writing – review & editing. EA: Writing – review & editing, Writing – original draft, Methodology, Supervision, Conceptualization, Project administration, Validation, Resources, Visualization. MP: Conceptualization, Methodology, Supervision, Validation, Project administration, Resources, Visualization, Writing – original draft, Writing – review & editing. JS: Conceptualization, Investigation, Methodology, Supervision, Validation, Writing – review & editing.
